# Does Addiction Have A Subject?: Desire in Contemporary U.S. Culture

**DOI:** 10.1007/s10912-021-09682-6

**Published:** 2021-03-06

**Authors:** Jaeyoon Park

**Affiliations:** University of California, Berkeley (Political Science), Berkeley, CA USA

**Keywords:** Desire, Addiction, Subject constitution, Foucault

## Abstract

This paper traces the emergence of a new figure of the desiring subject in contemporary addiction science and in three other recent cultural developments: the rise of cognitive-behavior therapy, the self-tracking movement, and the dissemination of ratings. In each, the subject’s desire becomes newly figured as a response to objects rather than a manifestation of the soul, measured numerically rather than expressed in language and rendered impersonal rather than individualizing. Together, these developments suggest a shift in the dominant form of the desiring subject in contemporary U.S. culture, one that breaks with the subject-form that Foucault theorized five decades ago.

This essay reads a recent shift in the medical-scientific notion of addictive desire as one site of a broader change in how subjective desire is figured and treated in U.S. culture. For most of the twentieth century, in basic research as well as clinical practice, the addict’s craving was understood as a collapsed or primitive form of human desire. Consider, for instance, how the physician and former U.S. drug czar, Ian Macdonald, portrays addiction in a scholarly monograph published in the mid-1980s. “Chemical dependency,” he writes, is akin to “natural dependency,” that is, to the psychic state of a child (1984, 30). Both conditions involve an obsession with “euphoria,” “distorted reality testing,” “impulsiv[ity],” “lack [of] the tools necessary for dealing with stress and intimacy,” and more (24-7). In both, the inability to resist one’s cravings for pleasure is the sign of a disordered or unintegrated psyche, and addiction is best understood as a kind of regression: “the 18-year-old chemically dependent child may be seen as a delinquent (which he is) or as depressed (which he is), or he may be seen as a 13-year old” (25). Fully grown, the addict becomes “an ‘adult’ who is amoral and without an ethical code except as it relates to personal desire” (98). Addictive desire expresses the psychic child in the adult body.

Macdonald was far from alone in propounding this view. In the 1970s and 1980s, this reading of addictive craving as indicative of psychic regression was circulated in premier medical journals as well as in popular media. Writing in the *Journal of the American Medical Association*, the psychiatrists Harold Kolansky and William Moore (1971) theorized addiction as a state of “ego decompensation,” in which mature psychic defenses give way to wild impulses and urges surging up from the id, while the physician Robert Kramer explained in a PBS documentary on drug abuse, screened at over eleven thousand town meetings on the night of its initial broadcast, that addicts are “18 going on 15 or 23 going on 16 in terms of their emotional, social, and intellectual development” (quoted in Kaiser 1983, 178). Earlier in the century, in one of the first major scholarly theories of addiction, the psychoanalyst Sándor Radó explained that the addict’s cravings ultimately issue from “a regression to the ‘original narcissistic stature’ of the ego” (1933, 18); the psychiatrist Lawrence Kolb, founding director of the federal government’s addiction research center at Lexington, Kentucky, wrote similarly in an anthology of his early papers that “chronic addiction…is invariably associated with emotional instability and immaturity,” and that “the present-day addict combines a number of traits which add up to his being an immature, hedonistic, socially inadequate personality” (1962, 4-5).

Yet in the last decade of the twentieth century, the view of addictive desire as primitive largely disappeared from medical-scientific discourse. Since then, medical science has taught instead that addictive desire is desire’s *maximum*, not its basest form. That is, addictive desire is not finally distinguished from other desires by its quality or its aim. Rather, desire is addictive when it is unduly intense or overwhelming. Thus the psychologist Jim Orford describes addictive desire as an “amplification” of ordinary desire and defines addiction itself as “strong attachment to an appetitive activity” (2001, 15). The strength of addictive attachment may be disabling, but it is neither childish nor crude: “the processes that give rise to strong appetitive attachment are normal ones” (29). Likewise, in another idiom, the neuroscientist Judith Grisel writes that addictive desire is produced by “normal brain functioning” in the face of unusually potent stimuli (2019, 47-8). Thus, if addictive desire is strong desire, ordinary desires are like modest addictions. The psychiatrist Isaac Marks writes that “normal biological cycles [such as eating and sex] are like chemical addictions in their mounting urge to do something that stops the urge, which increases again as time goes on” (1990, 1389). Or, as the neuroscientists James Burkett and Larry Young argue, “social attachment may be understood as a behavioral addiction, whereby the subject becomes addicted to another individual and the cues that predict social reward” (2012, 1). In this last instance, addiction is a paradigmatic form of desire.

This shift in the status of addictive desire—once figured as primitive, now as intense yet normal—has involved a more fundamental rethinking, in medical-scientific discourse, of what desire is. Twentieth-century addiction science presumed that desire is a form of psychic energy, a labile drive toward pleasure that can be organized into higher forms of object love and attachment. Macdonald cites such authorities as Freud and Erikson to elaborate a psychoanalytic view of human desire and its development: “man by nature seeks pleasure,” he explains, but under normal conditions the drive for pleasure is sublimated into “industry” and “achievement,” restrained by an “ethical code,” and regulated by “heterosexual identification” (1984, 97-9). This is how addictive desire could appear as undeveloped desire—aimed at pure pleasure, untrained, and wild. By contrast, in contemporary addiction research, desire is figured not as an inner drive that can be shaped and refined but rather as a motive formed in the subject as a response to stimulation by an object. Whether the desire is moderate or intense, a desire for drugs or a desire for love, it has the aim of procuring a previous gratification once again, and it is the direct result of that prior experience of gratification. Thus no desire is more or less refined or developed than any other, and what the subject desires is not a reflection of his or her psychic character. This is why, as we will see, addictive desire is not read for its quality in our day, but for its strength along with its consequences for social functioning; it is why addiction is imagined as akin to normal desire.

This essay studies this transformation in the figure of desire that frames and undergirds addiction science as part of a more general shift in how the desiring subject is imagined in contemporary U.S. culture. Over the past half century, many of us have learned to think about the Western subject of desire along the lines of Foucault’s *The History of Sexuality*. Foucault showed us how in Western modernity, the subject is increasingly formed as one whose desire, especially its sexual desire, constitutes its inner and identifying truth. That is, what this subject wants is an expression of who and what it is: desire is never contingent but manifests the state of one’s psyche or soul, and hence the way that one desires becomes the key to knowing what kind of person one really is. For this subject, I am my desire, or my desire betrays me; and this capacity of desire to individualize the subject exposes it to forms of regulation that seek to classify and correct “perverse” persons, as in Foucault’s well-known example of the production of “the homosexual” as a “species.”

It may be, however, that this modern figure that Foucault theorized no longer adequately captures the constitution of the desiring subject in our day. As I explain further on, by this I do not mean that the subject whose desire expresses the truth of his or her being has *disappeared* from contemporary culture but that it may no longer represent the *dominant* form of the desiring subject as it did in the historical period that Foucault studied, and that a genuinely novel figure of the desiring subject now appears in more and more venues of our society. This subject’s desire is not a manifestation of its innermost nature but a reflection of the stimuli it has formerly encountered, consumed, and thus developed a yearning for. In other words, this subject’s desire neither resides in nor reveals the composition of a psychic depth or soul; instead, it discloses what the subject has done and which objects have most gratified it, and thereby the power of those objects to instill desire at the site of the subject. My argument in this essay will be that desire is becoming, in our time, less and less something for the subject to confess about itself and increasingly impersonal, amenable to quantitative measure, and rooted in the object field. The reimagination of desire in contemporary addiction science will provide an entry point for exploring this historical phenomenon, but we will find that it is only one of several places where desire has been refigured in this way over the past forty years.

The next section of this essay closely reads the leading contemporary theory of addictive desire in order to mark its departure, not only from older accounts of addiction but also from the imagination of the desiring subject that Foucault saw as saturating the modern field of discourse and social practice. The essay then looks outside the domain of addiction and explores three apparently unrelated yet importantly convergent cultural developments in recent U.S. history to show that the desiring subject figured in contemporary addiction discourse now appears across a wide range of discursive and practical domains. In the emergence of cognitive-behavior therapy as the dominant mode of psychotherapy, in the rise of self-tracking as a popular mode of self-inquiry, and in the dissemination of ratings as a ubiquitous value-form, we will find the subject’s desire refigured along the lines of addictive desire: as best captured by quantitative metrics rather than by expressive language, as revealing the potency of objects rather than the identity of the subject, and as having its origin not within the desiring person but out there, in the object world. The essay concludes with some reflections on the stakes of this inquiry and with a set of further questions that this novel figure of the desiring subject leads us to pose.

## Reimagining addictive desire

To grasp how addictive desire is imagined in contemporary discourse, we turn to the literature of neuroscience. To be sure, the neuroscientific view does not exhaust how scholars and physicians view and treat addiction in our day, despite what its proponents may claim. And yet it does hold a privileged place within contemporary discourse; it is, for instance, the official outlook of the National Institute on Drug Abuse (NIDA), which funds most of the world’s scientific research on addiction, and even the most distantly alternative approaches to addiction are inevitably evaluated in relation to the neuroscientific view.^1^ Further, addiction neuroscience now provides the scholarly framing for most public-oriented explanations of how addictive craving is formed, such as the recent landmark Surgeon General’s Report on addiction (U.S. Department of Health and Human Services 2016) and the 2007 Emmy-winning HBO documentary, *Addiction*. Thus although it would be a mistake to imagine addiction neuroscience as the only existing discourse on addiction, it is to this discourse that we must look in order to grasp the most ubiquitous and widely accepted medical-scientific view of addictive desire in the present day.

The common understanding of addiction as a brain disease sometimes obscures the fact that neuroscientists understand addiction as a set of interrelated processes, which occur in three sequential stages (Koob and Volkow 2016). This matters, for our purposes, because addiction refers *both* to the development of an intense craving to use drugs (or to engage in some other addictive behavior) repeatedly and to the adaptations that occur in several regions of the brain as a result of repeated drug use, which reinforce without being identical with the core craving. My focus in this section will be on the development of craving, or the first stage of addiction, alone, for it is here that addictive *desire* (as opposed, say, to the heightened stress responses that also result from drug use and contribute to addictive behavior) can be directly explored. In particular, I want to examine the theory of incentive-salience attribution, which is now widely considered to be the leading account of addictive craving and its development. Grasping this theory will allow us to see how addictive desire has been reconciled with normal desire, and how the medical-scientific discourse on addiction has thereby come to circulate a novel figure of the desiring subject.

The theory of incentive-salience attribution was introduced by the neuroscientists Terry Robinson and Kent Berridge in 1993.^2^ It was intended as an advance on what were then the conventional answers to what they call “the question of addiction”—that is, why addicts cannot cease their addictive behavior—all of which reflected a general confusion about the nature of addictive desire (2000, S91). The first, and most traditional, answer was that addicts are driven by a desire for pleasure. The second answer, an inverse of the first, was that addicts are driven by a desire to relieve pain—in particular, the pains of withdrawal. The third was hardly an answer at all: many scholars, Robinson and Berridge note, simply defined drugs as positive reinforcers in the sense given to this term by B. F. Skinner and abandoned the task of *explaining* addictive behavior. On this last view, “people take drugs…because drugs promote drug-taking behavior”—a premise that could ground empirical research but was theoretically vacuous (S92).

If the radical-behaviorist answer to the addiction question was empty, the first two had foundered on the facts. It turns out that not just addictive behavior but also addictive craving tends to *intensify* over time, even as the pleasures of drug use steadily wane. Further, studies had shown that addictive cravings are often highest when withdrawal symptoms are lowest (that is, just before and during drug use) and that many drugs (like cocaine) have no withdrawal effects yet are highly addictive. For Robinson and Berridge, these empirical failures indicated that the very concept of desire operative in most explanations of addiction was flawed. If the first two answers failed, this was not because their focus on desire was misplaced (as the behaviorists claimed), but because they had made an unjustified assumption about desire. In particular, they assumed that desire is all about pleasure—that desire is always the desire *for* pleasure or its negative, that is, the desire to avoid pain. But what if desire and pleasure are only contingently related, so that addictive desire develops independently of pleasure and pain? What if addictive craving, at least in its initial development, has nothing to do with the subject’s affective state at all?

These are precisely the conclusions at which Robinson and Berridge arrived through their work on the mesolimbic dopamine system, long known to be the brain region directly targeted by drugs and other addictive objects, and long assumed (by scholars and the public alike) to be the site where pleasure is mediated in the brain. Against that longstanding assumption, Berridge and Robinson found that pleasure responses are not reliably affected by experimental manipulation of the mesolimbic circuits. Here, pleasure, or what Robinson and Berridge term “liking,” refers to the experience of “positive affective states” (2000, S93)—it is the feeling that washes over the subject in the wake of a dose of sugar or in the moment of a thrilling touch. Instead, Robinson and Berridge found that the region of the brain targeted by addictive objects mediates *desire*, or what they call the psychological experience of “wanting,” which is not incompatible with that of “liking,” but which has nothing to do in itself with affect or feeling (S105).^3^ Thus desire, in this theory, will refer not to a persistent striving for a maximum of pleasure but rather to the shifts in subjective perception and motivation that issue from one’s stimulation by certain objects.

The crux of Robinson and Berridge’s theory is that “the psychological process that leads to ‘wanting’”—in simple terms, the formation of desire—“involves the attribution of attractive salience to stimuli and their representations” (2000, S105). *Attribution* is a term of art here, and its crucial meaning becomes clear if we compare it to the psychoanalytic notion of *projection*. In projection, the subject *sends* or *throws out* a meaning or image that is primarily formed within the psyche of the projecting subject: the object is endowed with a value that is the subject’s own. Thus if my desire for you involves my projection onto you, then what I want in you is at least in part of my making, and this means that my desire is *personal*, inflected and informed by who I am and how my psyche has developed in the course of my own life history. By contrast, attribution is a process in which the subject does not *invest* the object with a personalized value or meaning but rather *registers* the potency of the stimulating object within its neural circuits and subjective perceptual field. In other words, attribution is a process through which the *object’s* own value—its capacity to stimulate the brains of human subjects—is established within the desiring subject. Here is how this works:

Attribution of incentive salience begins when the subject is first stimulated by an object that is capable of inducing desire (that is, that stimulates dopamine release within the mesolimbic system), either “via sensory receptors” (tongue, skin, eyes, etc.) or “more directly” (e.g., through the bloodstream or electrical brain stimulation) (1993, 291).^4^ As this stimulation occurs, the release of dopamine in the mesolimbic circuits inaugurates a process called “sensitization,” which corresponds to what I have called the subject’s *registering* of the object’s value within his or her physiology but also perceptual field. In Robinson and Berridge’s words, sensitization is a process that

transforms the sensory features of ordinary stimuli, or more accurately, the neural and psychological representations of stimuli, so that they become especially salient stimuli, stimuli that ‘grab the attention,’ that become especially attractive and wanted, thus eliciting approach and guiding behavior to the goal. (2000, S105)

In other words, when an object stimulates the mesolimbic circuits, its status for the subject shifts: no longer an *ordinary* stimulus, it becomes a *salient* stimulus, which means, according to the text just cited, a stimulus that “grabs” the subject’s attention and that “elicits” the subject’s approach. It is as if the object becomes highlighted—or in one of Berridge’s favorite metaphors, *magnetic*—within the experiential field of the subject. It is not that the object is associated with pleasure, or that the subject attaches a conscious or unconscious promise to the wanted object. Rather, what occurs is a transformation of the landscape that the subject inhabits in which the stimulating object grows bigger, more obvious, and more weighty, exerting a kind of gravitational pull on the subject.

As Robinson and Berridge note, certain contextual factors can “modulate the expression and development of sensitization.” For example, it appears that taking a drug at home results in a less powerful sensitization (the object is attributed a relatively smaller amount of salience) than taking that drug in an unfamiliar setting (S109, S97). However, the attribution of salience is primarily determined by the potency or “reward value” of an object. Further, it is cumulative: each time I am stimulated by the same object, the more sensitized I become to that object, and this means that all desire can potentially become addictive. In fact, addictive “craving,” in Robinson and Berridge’s glossary, is nothing more than the quantitative maximum of “wanting,” and a craving for drugs is simply the result of repeated experiences of sensitization—a repetition that is made likely by the fact that drugs are highly potent objects (they induce extreme amounts of dopamine on each occasion) and hence can take on a great deal of salience much more quickly than most rewards do. In any case, the craving for drugs is simply the result of the fact that “with repeated use drugs gradually become more and more attractive…and become increasingly able to control behavior” (2000, S99). It does not mean that addicts especially cleave to pleasure or that they endow drugs with a special promise or function that render the latter impossible to resist.

One can see, then, how addictive desire comes to resemble normal desire, even as they are not fully identified with one another. Berridge writes that “addiction is a pathological case,” both for its extremity and its debilitating consequences for social functioning and yet invokes addiction to demonstrate the dynamics of “incentive salience attribution,” which “is crucial to normal reward learning.” Incentive salience attribution is, according to him, how we form our desires for “sensory rewards” like “drugs and sex and food,” but its purview is wider than this: “human abstract and cultural and cognitive rewards tap into these same brain systems” (Berridge 2001, 257; Berridge 2016). Addictiveness is a potentiality in all these various domains because addictive desire is nothing more than what happens when the mechanisms of human desire-formation meet with intensely powerful rewards on repeat occasions, and it does not matter who the individual is or even what the specific object is—thus we see rising prevalence rates, in our day, of all kinds of diagnosed addictions, from gaming and internet addictions to work and shopping addictions and more.

I dwell on the theory of incentive-salience attribution at some length, not only for its inherent interest but also for the way it transforms, first, the way we *read* addictive desire—and indeed human desire more generally—and second, the relationship of the subject to its own desire. If desire is the result of attribution, and if it is neither bound up with a feeling nor expressive of a psychic state, the implication is not only that addictive craving cannot be measured by what the addict says about his or her experience of pleasure and pain but that craving can no longer be measured by what the addict claims to feel or think at all. This is, in part, because desire is mostly “pre-conscious,” but more importantly because desire is not made up of thoughts, images, and feelings; rather, it refers to the literal attraction of the subject by the object, the degree to which a given object “elicits approach” from the subject, in Robinson and Berridge’s phrase. Thus to understand desire, one does not need to capture and analyze the various personal meanings or affects with which it is invested; rather, desire is most clearly manifested in “behavioral measures”—the subject’s actual approach toward the object—and indeed Robinson and Berridge sometimes equate “wanting” with “goal-directed behavior” itself (2000, S100, S105).

This entails a radically new vision of how the subject relates to its own desire. Consider the divergence here from the two older frames of desire that Robinson and Berridge are aiming to contest. In the Freudian view, desire is a drive toward pleasure, organized by the constructions and defenses erected in and as the psyche, whose manifestations reveal how I manage my basic hedonistic nature and hence the kind of self I am. My desire is thus something for me to manage but also something from which I can learn something about myself: what I want gives expression to what I had to give up in my personal history, what I refused to give up, what I substituted for what I could not openly love, and so forth. In the Skinnerian view, my desire is nothing more than an epiphenomenon of my repertoire of learned behaviors, and intense feelings of desire are simply evidence of a circumstance which prompts a learned behavior that cannot, for some reason, be carried out (see Skinner 1976, 46-54). Against Freud, the theory of incentive salience suggests that desire is never an expression of the drive toward pleasure and that present desire is not shaped by a psychic history and does not invest the object world with meanings that I project from deep within me. Thus I am the site of my desire, but my desire says nothing about my past or my identity; it simply describes the way objects have taken on differential salience within my experiential field. But neither is my desire meaningless nor merely epiphenomenal; if properly measured, it has the capacity to show what I have encountered and how the world appears to me.

In both these ways, the theory of desire circulated by contemporary addiction science not only radically alters our thinking about addictive craving but also introduces a new figure into the history of the desiring subject’s constitution as an object of knowledge and power. Much of this history has been written by Michel Foucault. In his *History of Sexuality*, Foucault shows us that throughout the Middle Ages, the desiring subject appeared primarily within a juridical or social-customary frame. The subject’s desire was not directly examined for its moral quality or purity but rather assessed only on those occasions when it became manifest as conduct and then only in relation to the requirements of religious, civil, and traditional law. The question posed to the desiring subject was, *What have you done with your desire, and what, then, is your status before the law?* Yet in the early sixteenth century, the dominant framing of subjective desire underwent a dramatic shift. Desire became the individualizing truth of the subject—an expression of its identity and thus its personal secret—as it passed, in Foucault’s terms, from a juridical to a veridical frame. Now the question posed to the subject was, *What have you wanted to do, in your private thoughts and in your most intimate fantasies, and what does your desire reveal about the kind of person that you are?* As the key to one’s character and psychic composition, desire became the means by which the “I” could be diagnosed, pathologized, and exposed to techniques of reform, and not only to the force of legal punishment. In short, desire became something to be confessed, in Foucault’s well-known phrase: something to be revealed in expressive language, submitted to hermeneutic scrutiny, and given up only with reluctance, since concealment of one’s desire could now work as a modest shelter from the violence of regulation.

In Foucault’s narrative, this new framing of the subject and its desire was exemplified in the transformation of Catholic penance in the sixteenth century, when the confessant began to be probed in a newly intimate, exhaustive, and continuous way. But its rise to dominance only came about when the confessional figure of desire “lost its ritualistic and exclusive localization” within the religious domain. Over the course of the eighteenth century, Foucault writes, the confessional practice and framing of the desiring subject became “employed in a whole series of relationships: children and parents, students and educators, patients and psychiatrists, delinquents and experts,” and more (1978, 63). In medicine, psychiatry, education, criminal justice, and the family, desire was solicited, produced, and managed as the essential truth of individual subjects: “the truthful confession was inscribed at the heart of the procedures of individualization by power,” both in the sense that confession was actively practiced in a wide range of cultural domains and in the sense that the figure of the confessional desiring subject became presumptive in the dominant scientific and popular discourses of the age. Thus in nearly every significant domain of human existence, the modern subject comes to live and find oneself addressed in the image of the one who confesses a desire that reveals who one is: or, in Foucault’s famous phrase, “Western man has become a confessing animal” (59).

I want to suggest that the transformation of desire in contemporary addiction discourse forms part of a broader shift in the historical constitution of the Western desiring subject. For the subject figured by the theory of incentive salience, there is no distinction between higher and lower desires, noble and craven desires, and this means that the addict will no longer be identified as a primitive or immature subject by his or her desire; addictive desire reveals, rather, the potency of the objects that the subject has encountered and the history of repeated encounters between the subject and what it desires. In fact, this is a general implication of the incentive-salience theory: no desire bears the signature of one’s psychic character or identity. The point is not that desire now tells us *nothing* about the subject—to the contrary, it says much about what one has done, what one has engaged with, and what it is that moves one to action—but this desire does not comprise a “secret nature” of the self, something hidden away in an obscure depth and that may or must be “surfaced” (Foucault 1978, 60). In short, with the figure of desire that emerges in contemporary addiction discourse, desire no longer appears as something to be *confessed* but rather as something to be acted out and measured and as something that reflects the object world that the subject inhabits much more than the psyche or soul that desire once expressed. This development, I now want to argue, is not restricted to the domain of addiction and addictive desire alone. Just as for Foucault, a shift in the Catholic ritual of penance was part of a much more general transformation in the figure of the desiring subject, the new notion of “wanting” is a prism for the ascendance of a newly constituted desiring subject in our day.

## Convergent transformations

For Foucault, a dominant subject-form is not the collective spirit or ethos of an age but rather that specific figure of the human which is presumed and produced as the “correlate” of numerous culturally significant discourses and practices within a given time and place.^5^ What we are is neither fully determined by nor purely reflective of the historical moment in which we live, but we do each constitute ourselves and find ourselves constituted in the terms of certain privileged forms of knowledge that are authorized to name and describe the human; in the terms of those social practices such as medicine, law, and education that actively arrest, address, and handle us; and in the terms of scholarly and popular discourses that we ourselves engage as we seek to make sense of our lives and our being. When the terms of these discourses and practices converge on a consistent figure of the human—when each of them refers not to the same, but to a relatively shared or overlapping notion of what the human is and how it ought to be known and acted on—that figure takes on the status of a dominant subjective form, one that becomes the shared point of reference for so many of the cultural means through which subjects are described, identified, and managed.

Thus when Foucault wrote that Western man *has become* a confessing animal, he meant that the confessional subject had become the ubiquitous correlate of discourse and practice in the culture of Western modernity. This ubiquity is not the effect of a general social condition—it is not, for instance, the ideological reflection of a modern mode of production—but rather the result of myriad, sometimes interlinked but sometimes independent, historical processes. For instance, in Catholic penance the imagination of desire as emanating from the hidden folds of the flesh and bearing one’s secret is the result of technical reforms intended to make confession impracticable among the laity, thus increasing the prestige of the priests.^6^ In medicine, sexual desire becomes a bodily truth to be extracted from underneath the conscious protestations of the patient with the invention of a neurological technique that allows the doctor to induce unwilled yet telling effects at the site of the patient’s body.^7^ And in criminal justice, the offender is figured as the bearer of a dangerous and evil instinct toward criminal action beneath his or her legal status by the entry of expert psychiatric opinion into the courtroom, a move enabled by a single provision in the penal code that mitigates punishment for those who exhibit delirium.^8^ Each development is discrete and has its own causal history, but each establishes as its correlate—that is, each presumes and produces—a figure of the subject who bears a desire (sinful, sexual, criminal) that will be either hidden or confessed and that has the capacity to disclose the identity of the subject (wretched, hysteric, dangerous). As such developments multiply and interact, the confessional figure of the desiring subject begins to appear everywhere in modern culture—one encounters it in the church, in the clinic, in the courts and also in the school, in the family, in literature, and in the way we speak to ourselves and about ourselves to others. In this way, desire really becomes invested with our identity and our truth, and there is no way to live in the modern West without being subject to the demand to confess. Thus does Western man “become” a confessing animal, a creature portrayed and formed in the image of a confessional subject in every venue of its existence.

I rehearse these general points about Foucault’s view of subject-constitution in order to clarify the nature of the shift that I am charting in this essay. In claiming that the constitution of the desiring subject is now undergoing change, I do not locate this change in the transcendental conditions of our existence, and I do not mean to suggest that there has been a comprehensive displacement of the confessional figure of the subject. My claim is more precise and more modest: if we, as Western humans, *became* confessional animals in the course of modernity, in the sense that this subject-form became the correlate of most culturally significant discourses and social practices, then it would seem that the extent to which we *are* confessional animals is on the wane in our late-modern day. This is not because our essence is changing or because of a generalized social transformation such as a mutation in the logic of capital—indeed, I do not think that we can identify a single *cause* for the waning of the confessional subject at all. Rather, if we are no longer, or less and less, constituted as confessional animals, this means that such a figure of the subject no longer functions as the ubiquitous correlate of the dominant discourses and practices in contemporary Western culture. We have seen how this transformation occurs in the context of the scientific discourse on addiction: in tracking a localized theoretical shift, we saw how the figure of the desiring subject circulated by addiction science shifts from a confessional form, in which one’s desire reflects one’s psychic character, to one in which one’s desire is a response to gratification by objects and in which desire is thoroughly depersonalized, rendered incidental to the personal constitution or identity of the desiring subject. And as desire loses its symbolic status, that is, its capacity to bear and condense a hidden meaning about the subject, we see a shift in its proper medium of expression: no longer captured and read in the form of apostrophic language, desire becomes a fact and magnitude to be charted and measured in the form of behavioral metrics.

I want to suggest that this shift in the correlative figure of addiction discourse converges with recent developments in several other venues of U.S. culture, and that this convergence may be read as a sign that the Western human is, today, becoming something other, or perhaps simply more, than a confessional animal. Even if the confessional subject is still presumed and produced in our world, it is, I think, neither a ubiquitous nor unquestioned correlate of discourse and social practice, and it may be that a new dominant figure is coming into view. Let us explore just three recent cultural developments convergent with the changes in contemporary addiction science, each of which has its discrete historical conditions yet produces a subjective figure whose desire is, if not addictive, *akin* to addictive desire in its depersonalized, object-oriented, and measurable character. These developments are: the waning of psychoanalytic therapy and the subsequent dominance of cognitive-behavior therapy; the emergence of self-tracking as a popular practice for producing self-knowledge; and the dissemination of ratings as a technique for the expression of value.

## Psychotherapy

The psychoanalyst’s clinic was one of the main sites, in Foucault’s view, for the production of the subject as a confessional animal in modern Western society. The ritual of confession might well have been a description of free association and analysis, and it was Freud who argued that “a person’s ‘character’ is built up to a considerable extent from the material of sexual excitations and is composed of instincts [*Triebe*] that have been fixed since childhood, of constructions achieved by means of sublimation, and of other constructions, employed for effectively holding in check perverse impulses which have been recognized as unutilizable” (1953, 239). Thus, the decline of psychoanalysis as a dominant therapeutic framework in the late twentieth century and the rise of cognitive-behavior therapy to near-hegemony in its wake marks a major event in the history of the desiring subject. The correlate of most psychotherapeutic knowledge and practice is less and less likely to be the subject whose desire discloses a character in our day. The question is, what correlative figures of the human and its desire are formed by the discourse and practice of CBT?

Consider here the reflections of Aaron Beck and Albert Ellis, both of whom are generally recognized as the theoretical founders of CBT. Beck’s search for a new therapeutic theory and approach emerged from his dissatisfaction with “the psychoanalytic unconscious motivational and therapeutic method.” In particular, Beck came to reject the idea that psychopathology is the result of a specific instinctual-developmental history and sought to explain patients’ symptoms on the basis of present and directly observable phenomena. “Differentiating the cognitive from the psychoanalytic approach,” he writes, meant “focusing the treatment on present problems, as opposed to uncovering hidden traumas from the past, and on analyzing accessible, rather than unconscious, psychological experiences” (Knapp and Beck 2008, S56).

The psyche is not, Beck argued, a hidden and enduring organization of primary drives but rather a particular way of filtering, interpreting, and cognizing lived experience. It comprises not fixation points, constructions, and defenses but rather “*idiosyncratic cognitive structures*” or “schemas” that make sense of the “kalideoscopic array of stimuli” which are the raw material of subjective experience (Beck 1964, 561-2). Each of one’s schemas—which are learned or internalized over time and of which there are many in any individual psyche—functions as a “major premise,” in Beck’s metaphor, in the sense that it combines with an “external configuration” of stimuli, or a “minor premise,” and thus gives rise to conscious thought and emotion, which is the “conclusion” and the substance of one’s experience (563). All pathological symptoms, according to Beck, can be traced back to disturbed cognitions or feelings that are, in turn, the result of false schemas—an incorrect premise about the world, such as a belief that unfamiliar conditions necessarily harbor danger—or of a persistent misapplication of a true schema to inappropriate stimuli. Hence symptoms are like “inaccuracies, misinterpretations, and distortions,” and the therapist’s task is to identify “a recurrent erroneous conclusion”—an overly negative emotion, or depressing idea—from which “one can infer the content of the idiosyncratic schema” (563). The method of cure is then to draw the patient’s attention to this schema and help to eliminate it through conscious development of alternative schemas.

Ellis, too, developed his variant of CBT as a rejection of the premises of psychoanalysis. In Ellis’s idiom, psychopathological symptoms can be traced back to certain “underlying beliefs” or “axioms” that distort a patient’s experience of reality (1975, 24-6). Disturbed patients “*assume*” a certain premise about the world, “*look* for the ‘facts’ to prove [the] premise,” and then arrange these into “unvalidated sentences” about themselves and about their environment. The root cause, then, is the presence of problematic “definitional concepts” in the patient’s psyche, and Ellis goes so far as to claim that “all human disturbances seem to be of the same definitional nature” (26-7). As for Beck, symptoms are for Ellis the result of a false or overly active premise in the psyche, which generate the unrealistic thoughts and feelings that undergird such things as compulsions, debilitating fears, and other neuroses.

How, then, will the desiring subject appear in the theory and practice of CBT? It should be clear that desire loses its capacity to disclose the character of the psyche, not only because the psyche is no longer integrated as a personality but also because desire is no longer privileged as the substance and truth of psychic formation. Rather, it is one of several subjective phenomena—alongside conscious thoughts and other feelings—that do not originate within the subject, but rather emerge as the composite production of the learned frameworks through which one makes sense of the world and of the various objective stimuli that these frameworks meet. Desire is thus rendered impersonal in the sense that one’s desire does not reveal one’s developmental history or characterological tendencies; it reflects a set of articulate premises that may be deeply ingrained but which are subject to major revision and even replacement without a deep probing, let alone comprehensive change, of one’s personality. Desire is not read, by the CBT therapist, as a code to be deciphered for meaning but rather as one example of the “stereotyped or repetitive patterns of conceptualizing” that underlie psychopathology, and this means that desire does not need to be surfaced in an interpretive ritual (Beck 1964, 562); rather, it can simply be reported or observed, or can even be left out of the therapeutic exchange altogether. The subject of cognitive-behavior therapy is not, as in psychoanalysis, a subject of desire above all, and the desire of this subject is not a secret: it is neither hidden nor revealing when exposed; it is not the key to an identity. In these ways, the rise of CBT converges with the collapse of distance between addictive and normal desires: in both cases, desire is de-personalized and de-symbolized, made into a response to the world rather than an expression of interiority, and hence into something not to be confessed.

## Self-tracking

Over recent decades, the figure of desire as a confessable secret has lost its dominant status as well as the correlate of techniques for the production of self-knowledge. Increasingly, knowing oneself takes place through the generation of data about oneself in contemporary U.S. culture. In the late 1980s, workers in the computer industry produced the first experimental technologies for what is now called self-tracking; since then, both the equipment and habits of self-knowledge by means of tracking have become common in schools, workplaces, and everyday experience. One can purchase monitors and trackers for physical activity, work productivity, learning outcomes, and consumption habits in retail as well as app stores, and self-tracking has become a feature of subcultural groups ranging from San Francisco techies to so-called “health goths” (Neff and Nafus 2016, 4). Personal health remains the only domain in which a large majority of Americans now track themselves daily, but self-tracking as a cultural phenomenon is not restricted to any single domain. In her analysis of popular media coverage of self-tracking in recent decades, the sociologist Deborah Lupton finds that “by 2012, news articles represented quantified-self practices as growing in popularity and becoming not only an important feature of health promotion but a part of everyday life, as a way of maximizing productivity and happiness as well as health” (2016, 15).

To be sure, self-tracking does not obviate alternative means of self-knowing, in the way that CBT claims to have rendered psychoanalysis not just inferior but obsolete. And yet what is significant for my purposes here is how the subject is figured where and when it is the object of self-tracking. To see this, I want to consider the discourse of the Quantified Self community, an online organization with in-person regional chapters that is mostly responsible for elaborating the theoretical framework of self-tracking, and which has functioned, according to Lupton, as “the public face” of self-tracking as a cultural movement (12). In particular, let us examine the point and premise of self-tracking as a mode of “self-knowledge through numbers” as it is elaborated in a 2010 article in the *New York Times Magazine* authored by Gary Wolf, co-founder of Quantified Self and the leading promoter of this novel practice.

According to Wolf, the development of self-tracking over the past forty years is not just the popularization of a marginal pastime. It is, rather, the culmination of a centuries-long historical process. Modernity itself, he argues, is an epoch in which “the fetish for numbers” takes hold of an ever-increasing range of human experience (2010). This fetish first established itself “in science, in business, and in the more reasonable sectors,” as quantitative metrics became both the gold standard of evidence and the proper medium of communication in each sphere. From there, the fetish for numbers conquered nearly every other field, until “only one area of human activity appeared to be immune”: “personal life.” For most of the twentieth century, “the imposition, on oneself or one’s family, of a regime of objective record keeping seemed ridiculous.” This domain alone remained under the sway of the fetish for language, so that if “a journal was respectable,” “a spreadsheet was creepy.” In our time, this has changed. Today, “numbers are infiltrating the last redoubts of the personal,” as ordinary people now render in numbers their “sleep, exercise, sex, food, mood, location, alertness, productivity, even spiritual well-being.”

More than a shift of technology, the move from language to numbers changes the self to be known. “A hundred years ago, a bold researcher fascinated by the riddle of human personality might have grabbed onto new psychoanalytic concepts like repression and the unconscious,” and then rendered the human itself in linguistic form: “these ideas were invented by people who loved language” and hence held that “the road to knowledge [about the self] lies through words.” This meant that “even as therapeutic concepts of the self spread” beyond the sphere of psychoanalysis “they retained something of the prolix, literary humanism of their inventors.” One saw the self in literary terms, as a meaning to be expressed or disclosed in language. This entire vision is what self-tracking displaces. “Trackers are exploring an alternate route. Instead of interrogating their inner worlds through talking and writing, they are using numbers.” And against the literary self, “they are constructing a quantified self.”

What is the desire of the quantified self? To begin with, it is not a secret: “When we quantify ourselves, there isn’t the imperative to see through daily existence into a truth buried at a deeper level.” But that does not mean that desire is easily seen. For desire, just like the rest of human interiority, is not an obvious fact or visible in a single instance. Rather, it is something to be observed over time, something indicated much more by what one does and how one responds to certain conditions, rather than by what one may profess or even perform at any given moment. Consider the example of one self-tracker featured on the Quantified Self website who wanted to “definitively prove my love for my husband and not somebody else” with a tracking experiment (Jonas 2016). Years into her marriage, she knew what she consciously felt yet sought proof of her desire in numbers. She produced a data set tracking her behavior retroactively by converting all her chat messages from her computer into a searchable plain-text database. She searched, in this data, for what she calls a “sign of love,” not by probing what she said for what she meant but by tracking her patterns of verbal behavior. She considered how often she typed her husband’s name, relative to other words; how often and what times of day she wrote to her husband, relative to colleagues and friends; the number of words that were unique to her chat history with her husband; and so forth. Each would signify love, not by surfacing what lay in the tracker’s heart but by revealing intense or otherwise privileged forms of engagement. Desire *is*, in this experiment, a particular pattern of behavior; the task is “to see if there was love *present in my chatting behavior*” (my emphasis).

The point here is not that this self-tracker *believes* that love is reducible to behavior. It is rather that the figure of the self produced as the correlate of self-tracking is one whose interiority is not a psychic depth but rather habitual patterns of behavior charted over time. In Natasha Dow Schüll’s phrase, in the techniques of self-tracking, “bits and moments, accumulating into habits, rhythms, and tendencies, are the ‘stuff’ of the self,” and this is as true of one’s desire as it is of one’s capacity for productivity, one’s attentiveness to one’s children, and one’s mental health (2019, 33). The premise, and the promise, of self-tracking is that these aspects of our inner worlds will be disclosed, not by what we say, which is always liable to error and distortion, but by what we do, and especially by what we do as it gathers into patterns over time. As self-disclosure for the sake of self-knowledge increasingly takes the form of the tracked record, rather than the first-person narrative or diaristic revelation, the practice of confession retreats from another venue of social experience, and the subject as confessional animal accordingly wanes as its correlate. Moreover, the subject’s desire as produced by the techniques of tracking will resemble, and thus reinforce, the figure of desire whose emergence we are charting—a desire that belongs to without betraying the subject, a desire that is acted out much more than hidden away, and a desire that is capturable by metrics and not only, indeed not well, by the medium of expressive language.

## Rating

One of the main features of what many have called our financialized contemporary world is the ubiquitous use of ratings as a measure of value. Today, ratings are produced and consulted as a primary metric for expressing the value of consumer goods, financial products, and commercial experiences but also of such things as beauty and art, education, intellectual products, and the standing, trustworthiness, and future promise of individual persons. It may seem unintuitive to think of ratings as an expression of *desire*, as well, but consider that the value captured by the value-form of ratings is the capacity of the rated object to attract investors, both now and in the future, and hence can also be understood as the *attractiveness* of the rated object. The rating, in short, reflects past and present desire for what is being rated, as well as the likelihood that the rated object will retain its ability to attract desire in the future—a fact made especially plain, as we will see, in ratings of physical or intimate attractiveness.

But let us ask, once again, what *kind* of desire is capturable in the rating? Revealing here is the ongoing trend among online platforms to replace star-rating systems with a binary system in which the object is rated with a “thumbs-up” or “thumbs-down.”^9^ Consider, for instance, the widely publicized decision by Netflix to make this change in 2017. “Five stars feels very yesterday now,” insisted Todd Yellin, a senior executive at the company (Goode 2017). The main problem with the star-system, he explained, was that users tended to employ ratings as a form of self-expression, that is, as a way of indicating the kind of content that they would want to have associated with their identity or personality. Yet the purpose of ratings is to demonstrate the value of new products, meaning their actual capacity to attract the attention and engagement of users. Yellin explained: “We’re spending many billions of dollars on the titles we’re producing and licensing, and with these big catalogs, that [magnitude] just adds a challenge. Bubbling up the stuff people *actually want to watch* is super important” (my emphasis). For ratings to achieve this “bubbling up”—that is, for them to capture the real value and attractiveness of new titles—they cannot be used to show what one wants others to *think* that one wants to watch. “What’s more powerful” in this regard, asks Yellin: “you telling me that you would give five stars to the documentary about unrest in the Ukraine; that you’d give three stars to the latest Adam Sandler movie; or that you’d watch the Adam Sandler movie 10 times more frequently?” “What you do versus what you say you like are different things,” Yellin concludes, and it is the former that the implementation of the binary system is supposed to help the ratings capture and expose.

Notice that in the course of Yellin’s speech, three things become importantly confused, substituting for one another. First, ratings are intended to convey a users’ *desire*, what they “want to watch”; but second, this desire is construed as equivalent with what they *do* watch, rather than what they say or think about themselves; and third, what their desire discloses is the probability of their watching, in the future, what they are watching now. Thus desire is construed, at least by the Netflix rating, as equivalent with behavior and actual investment of attention, which is why the effectiveness of the rating system can be checked by comparison to measures of past activity: according to Yellin, one reason for the company’s decision to shift to the binary system was that its results better aligned with what Netflix could already glean from analyzing users’ browsing histories. And this desire is employed as a measure, not of who the audience is or what they want as a collective subject but rather of what the value of the object is—its capacity to attract and hold the attention of viewers.

Obviously, a Netflix rating is not the same thing as a credit rating, a college rating, or a swipe across the Tinder screen. But in each case, the rating measures and in doing so *figures* our desire as a response to an attractive object much more than an expression of personality. Desire is, in other words, a reflection of the *desirability* of the object, not something formed within and specific to me. The Tinder rating is an especially good example of this. Generally, users cannot view their own personal ratings, but the company does assign one to each user in order to enable pairings among similarly “desirable” individuals. Here is how the system works, as explained by a journalist: “You rose in the ranks based on how many people swiped right (‘liked’) you, but that was weighted based on who the swiper was. The more right swipes that person had, the more their right swipe on you meant for *your* score. Tinder would then serve people with similar scores to each other more often, assuming that people whom the crowd had similar opinions of would be in approximately the same tier of what they called ‘desirability’” (Tiffany 2019). Note that here again, what one desires is registered by what one does—one’s “opinion” of desirability is not spoken, but “swiped”—and what the rating reveals is the desirability of the rated object, not the identity of the swipers, who meld into the figure of a “crowd.” What one wants is not a secret and it is not personal; it is evident in one’s behavior and incited by the valuable object.

As ratings become an ever-more dominant form of expression for desire and desirability, these latter lose their indexical relation to personalities and become exchangeable across *any* subject that encounters the rated object. Indeed, as we have seen, the desire registered by a rating is *predictive* of the desire of another subject, whoever he or she is, since my desire is nothing more than how this object engages me or leads me to act: the desire that I experience is the desire of a crowd; it is impersonal and on the surface, rather than a sign of what moves me as a result of my constitution or my history. This figure of desire becomes legible wherever ratings take hold as a major practice. Consider one final example: the philosopher Michel Feher’s recent argument that ratings might become a prominent form of political agency in this century. Taking inspiration from the divestment campaign waged against the Keystone Pipeline, Feher suggests that “in a speculative age,” battles over political power must aim at “altering the conditions of accreditation” much more than the conditions of wage-labor (2018, 56-7). This means that we must exercise a “rated agency,” that is, our capacity “to speculate with other like-minded investees on what assets should be recognized as appreciable and thus on who deserves to be called creditworthy,” and to do this, tangibly, through “the collection of information and the publication of ‘opinions’ on the risks of an investment” (209, 84). Feher is compelling, in my view, but note what happens to the political tract in this exercise of agency: we give voice to our desire to divest from organizations and projects that are exploitative or that work against environmental sustainability, but this desire is voiced as a measure of the *undesirability* (the non-appreciability or low creditworthiness) of the object, rather than as an expression of who we are as a people. The subject of this desire is a faceless subject, one that does not confess its desire but seeks to publish it, as a reflection of what it rates much more than who is rating. The desire of the rating subject thus converges with that of the addict-subject, the therapeutic subject, and the self-tracking subject in its impersonality, its responsiveness to the object, and its capacity to be observed and registered in numerical metrics. In each of these ways, this desire diverges from that of the confessional subject, and it does so, I hope to have shown, in a growing range of venues in contemporary cultural experience.

## Conclusion

We live in “a singularly confessing society,” Foucault insisted, imagining that one could not escape being formed as a subject that confesses its desire in the modern West: “One confesses—or is forced to confess. When it is not spontaneous or dictated by some internal imperative, the confession is wrung from a person by violence or threat; it is driven from its hiding place in the soul, or extracted from the body” (1978, 59). He could not have known that three decades after his death, the confessors would find themselves frustrated, not by the resistance of confessants, but by the fading of the guarded soul and withholding body. The psychoanalyst Christopher Bollas writes, in a recent book, that what he once diagnosed as the illness of “normopathy,” a term that he borrowed from the analyst Joyce McDougall, now characterizes a ubiquitous type of patient that has been “on the rise” in the Euro-American world since the “third quarter of the [twentieth] century.” The normopath has many symptoms, in Bollas’s description, but the “fundamental characteristic” of this figure is “a lack of interest in subjective life” that constitutes a kind of immunity to interpretive analysis (2018, 42-3). One exemplary patient responds, to Bollas’s interpretation of her persistent desire to become indispensable: “You got it… That’s brilliant—thanks so much,” as if the analyst were less a diver of psychic depths than “a good auto mechanic or computer expert” (64). There is no question of forcing the confession here: the problem is not the lack of an internal imperative but the fact that the subject has nothing to confess, and the admission of her desire is utterly meaningless, disclosing nothing because she is hiding nothing, no secret and no soul that this secret would express. Indeed, McDougall first theorized the normopathic patient in order to describe a sort of person “becoming ever more frequent in today’s practice.” Tellingly, her second name for this sort of person was “the anti-analysand” (1992, 213, 215).

Let us read this stumble in the life of psychoanalysis, not as a sign of its death or even as an indication that its correlative figure of the subject has disappeared—after all, there *is* a vibrant life of psychoanalysis, even as it has come to be lived on the cultural margins—but as suggesting that the Western human is not, or not so much, constituted as a confessional animal in the present day. By this I mean not that the shared spirit of the present moment has mutated away from that of the past, but that the contemporary cultural landscape features fewer discourses and practices that figure and form us in the image of the confessional animal, and that we are thus constituted and constitute ourselves in this image on fewer occasions and with less regularity in our day. Think of the shift that I am charting here less as the replacement of one species by another, and more as the diversification of the landscape: our world is no longer the world of the confessional animal alone, and may further be a world in which the confessional animal is less likely to thrive. This is because the kinds of subjects that we are depends on the social practices, institutions, and forms of knowledge that *make* us into a certain kind of being; when this making ceases or wanes, we become addressed, handled, and figured in terms that make us differently, and this means that our constitution shifts and morphs with the social world in which we live. My claim in this essay has been that our social and cultural world has importantly shifted in the past forty years, so that we are now constituted newly as desiring subjects—as subjects whose desire is not a hidden secret or a code to be deciphered, and hence as subjects without the bodies and souls that such desires once expressed. At this moment, it remains unclear what the body and soul are increasingly becoming, and to chart this becoming is the task that Foucault’s work now leaves to us, to be pursued through an examination of the dominant discourses and practices that address or handle our desire, and of the points of convergence among their correlates. This is surely one central reason why attending to developments in medical science holds a vital importance for humanistic inquiry today.
